# Abscisic Acid Induced Changes in Production of Primary and Secondary Metabolites, Photosynthetic Capacity, Antioxidant Capability, Antioxidant Enzymes and Lipoxygenase Inhibitory Activity of *Orthosiphon stamineus* Benth.

**DOI:** 10.3390/molecules18077957

**Published:** 2013-07-05

**Authors:** Mohd Hafiz Ibrahim, Hawa Z. E. Jaafar

**Affiliations:** 1 Department of Biology, Faculty of Science, University Putra Malaysia, 43400 Serdang, Selangor, Malaysia; 2 Department of Crop Science, Faculty of Agriculture, University Putra Malaysia, 43400 Serdang, Selangor, Malaysia; E-Mail: hawazej@gmail.com

**Keywords:** abscisic acid, *Orthosiphon stamineus*, plant secondary metabolites, photosynthetic performance, antioxidant capabilities, antioxidant enzymes

## Abstract

An experiment was conducted to investigate and distinguish the relationships in the production of total phenolics, total flavonoids, soluble sugars, H_2_O_2_, O_2_^−^, phenylalanine ammonia lyase (PAL) activity, leaf gas exchange, antioxidant activity, antioxidant enzyme activity [ascorbate peroxidase (APX), catalase (CAT), superoxide dismutase (SOD) and Lipoxygenase inhibitory activity (LOX)] under four levels of foliar abscisic acid (ABA) application (0, 2, 4, 6 µM) for 15 weeks in *Orthosiphon stamineus* Benth. It was found that the production of plant secondary metabolites, soluble sugars, antioxidant activity, PAL activity and LOX inhibitory activity was influenced by foliar application of ABA. As the concentration of ABA was increased from 0 to 6 µM the production of total phenolics, flavonoids, sucrose, H_2_O_2_, O_2_^−^, PAL activity and LOX inhibitory activity was enhanced. It was also observed that the antioxidant capabilities (DPPH and ORAC) were increased. This was followed by increases in production of antioxidant enzymes APX, CAT and SOD. Under high application rates of ABA the net photosynthesis and stomatal conductance was found to be reduced. The production of primary and secondary metabolites displayed a significant positive relationship with H_2_O_2_ (total phenolics, r^2^ = 0.877; total flavonoids, r^2^ = 0.812; *p* ≤ 0.05) and O_2_^−^ (total phenolics, r^2^ = 0.778; total flavonoids, r^2^ = 0.912; *p* ≤ 0.05). This indicated that increased oxidative stress at high application rates of ABA, improved the production of phytochemicals.

## 1. Introduction

*Orthosiphon stamineus* Benth from the Famiy Lamiaceae is locally known as Misai Kucing in Malaysia and Kumis Kuching in Indonesia. This plant is commonly found in tropical countries such as Thailand, Indonesia, Philippines, Brunei and Malaysia. Although found growing in the wild, the plant is also used as an ornamental plant. *Orthosiphon stamineus* is traditionally used in Southeast Asia as an herbal tea or diuretic to treat kidney disorders, abdominal pains, gout, fever, hypertension, hepatitis, jaundice and diabetes [1]. Moreover, it has been scientifically proven that *O. stamineus* exhibits a range of pharmacological properties such as anti-inflammatory, anti-oxidant, anti-bacterial, anti-angiogenic properties and has hepatoprotective effects [2]. *Orthosiphon stamineus* contains more than 20 phenolic compounds, including nine caffeic acid derivatives, including rosmarinic acid and 2,3-dicaffeoyltartaric acid, two flavonol glycosides and nine lipophilic flavones [3]. The main components of *O. stamineus* leaves are polyphenols (caffeic acid derivatives and the polymethoxylated flavonoids) [4]. The therapeutic effects of *O. stamineus* have been ascribed mainly to its polyphenols which form the most dominant constituents in the leaf. These constituents have been reported to be effective in reducing oxidative stress by inhibiting the formation of lipid peroxidation products in biological systems [1].

Phenolics and polyphenolics compounds are characterized by an aromatic or phenolics ring structure. These compounds are include the flavonoids, phenolics acids and lignans. Phenolics compounds are located in vacuole, they are found in free from or linked to carbohydrates (glucose, galactose, rhamnose, mannose, rutinose) and tend to be soluble in water or organic solvents [5].These compounds are well recognized to be a good treatment against chronic illnesses such as cancer and cardiovascular diseases [6]. The concentration of phenolic compounds in fruits and vegetables is regulated by genetic, environmental, physiological and chemical factors such as temperature, light, rainfall, soil, chemicals and plant growth regulators [7]. Various agronomic strategies such as alteration of environmental conditions, water management, grafting of plants, application of elicitors, stimulating agents and plant activators have been employed to enhance the biosynthesis of phenolic compounds in fruits and vegetables [8]. One popular strategy is the application of ABA, a plant growth regulator involved in various physiological processes including colour development [9].

Studies have shown that exogenous ABA application affects growth and phytochemical content, and is of particular interest in food and nutritional sciences as it increases the nutritional value of several fruits and vegetables. It has been documented that ABA can induce the expression of antioxidant genes and enhance the capacity of antioxidant defense systems, including enzymatic and non-enzymatic constituents [10]. Meanwhile, since ABA causes oxidative stress in plants, a high concentration of ABA induces excessive generation of active oxygen species (AOS) and leads to oxidative damage in plant cells [11]. In a recent study involving greenhouse red and green leaf lettuces, exogenous ABA application significantly increased the anthocyanin content in red leaf lettuce along with the contents of chlorophyll b and total carotenoids in the green leaf lettuce when compared to the controls [12]. Also, exogenous ABA was found to stimulate anthocyanin biosynthesis and increase the content of phenolic compounds in Noble muscadine grapes [13]. 

ABA also increased the generation of H_2_O_2_ and O_2_^−^ [14,15]. The free radicals such as ROS, including hydroxyl radicals, superoxide anions and hydrogen peroxide, play an important role in promoting tissue damage in living organisms. They may lead to cell damage through membrane lipid peroxidation and DNA mutations and as a consequence of that diseases such as cancer may develop [16]. Moreover, ABA is known to enhance activities of antioxidant enzymes such as superoxide dismutase (SOD), ascorbate peroxidase (APX), glutathione reductase (GR), and catalase (CAT) in plant tissues [17,18,19]. As H_2_O_2_ is relatively stable and diffusible through membranes, it is considered a general signal molecule [20]. Tsai and Kao [21] demonstrated that H_2_O_2_ was involved in ABA-induced activities of APX and GR in rice roots. Water stress-induced ABA accumulation has been shown to trigger increased generation of H_2_O_2_, which in turn led to the up-regulation of secondary metabolites and antioxidant enzyme activities in maize leaves [22,23].

The antioxidant activity of phenolic compounds was found to be mainly due to their scavenging and redox properties through neutralizing and quenching of free radicals. Since the application of ABA has been proven to enhance the production of phytochemicals, It is hypothesized that the use of exogenous ABA might also enhance the production of specific secondary metabolites and increase antioxidant activity in *Orthosiphon stamineus*. Thus, the primary aim of the present research was to investigate the effects of ABA elicitation on the accumulation of primary and secondary metabolites and antioxidant activity in *O. stamineus,* which would be beneficial to the Malaysian herb industry. The study was also designed to assess the impact of foliar ABA applications on the leaf gas exchange, the secondary metabolic enzymes (PAL), the antioxidative enzymes (SOD, CAT and POD) and lipoxygenase (LOX) inhibitory activity.

## 2. Results and Discussion

### 2.1. Total Phenolics and Flavonoids Profiling

Accumulation of total phenolics and flavonoids in *O. stamineus* were influenced by ABA levels (*p* ≤ 0.01; Table 1). Generally total phenolics content was highest in the leaves followed by roots and stems. As the plants received higher ABA levels (2 > 6 µM) the production of total phenolics and flavonoids was enhanced. The total phenolics content in the leaf (with ABA at 4 µM, 6 µM, 0 µM), roots (with ABA at 6 µM, 4 µM, 2 µM, 0 µM), and stems (with ABA at 6 µM, 4 µM, 2 µM, 0 µM) were 3, 6, 35, 42, 44, 46, 55, 120, 168, 180, and 218%, respectively. These values were low compared to the leaf receiving 6 µM ABA that registered 4.21 mg gallic acid g^−1^ dry weight. Total flavonoids content followed the same trend as total phenolics where the highest total flavonoids was observed in the leaf receiving 6 µM ABA that registered 2.12 mg rutin g^−1^ dry weight, and the lowest was in the stems with the control treatment (0 µM) that contained only 0.52 mg rutin g^−1^ dry weight. The increase in the production of total phenolics and flavonoids with increasing ABA has also been observed in *Vitis rotundifolia* [13] and *Vitis vinifera* [24]. Wang *et al. *[25] observed that as the level of foliar application of ABA increased, the production of soluble sugar was enhanced in *Atractylodes macrocephala*. Carbohydrates are basic compounds required to produce phenolic compounds through the shikimic acid pathway where extra carbohydrates derived from glycolysis and the pentose phosphate pathway are converted into aromatic amino acids. Previous studies by Shui *et al. *[26] showed that an increase in secondary metabolites was related to the balance between carbohydrate source and sink; the greater the source-sink ratio, the greater the production of secondary metabolites that might occur. This was also reported by Guo *et al. *[27] that found an increase in sucrose content corresponding to the enhanced production of ascorbic acid, glucosinolates, sulforaphane, anthocyanins, total phenolics and increased antioxidative activities in broccoli sprouts. This was supported by source-sink hypotheses (carbon nutrient balance hypothesis) [28] and growth-differentiation balance hypothesis [29] that assume that increase in carbon accessibility that is accumulated in total non-structurable carbohydrate (TNC) would increased the production of carbon based secondary metabolites (total phenolics and flavonoids) when the provided carbon amounts exceed growth requirements [30]. This suggests that the increase in the production of total non-structural carbohydrates might up-regulate the production of total phenolics and flavonoids with the application of ABA in the current study. This fact is supported by the high significant correlation coefficients of total phenolics (r^2^ = 0.971; *p *≤ 0.05) and total flavonoids (r^2^ = 0.953; *p* ≤ 0.05) with soluble sugars (Table 2). The present results indicate that exogenous application of ABA can enhance the production of carbon based secondary metabolites like total phenolics and flavonoids. In previous studies the application of abscicic acid have been shown to improve the production of tanshinone in *Salvia miltiorrhiza* [31], chlorogenic acid in *Phragmites communis* [32], and terpenoids in *Cannabis sativa* [33], thus indicating the importance of ABA in increasing the production of secondary metabolites in plants.

**Table 1:** Impact of abscisic acid on total phenolics, flavonoids and soluble sugars produced in different parts of *Orthosiphon* *stamineus*.

ABA (µM)	Parts	Total Phenolics (mg g^−1^ gallic acid dry weight)	Total Flavonoids (mg g^−1^ rutin dry weight)	Soluble sugar (mg g^−1^ sucrose dry weight)
	Leaves	3.11 ± 0.27 ^c^	1.47 ± 0.21 ^c^	79.12 ± 11.21 ^d^
0	Stems	1.32 ± 0.02 ^l^	0.52 ± 0.02 ^g^	40.23 ± 8.98 ^l^
	Roots	2.71 ± 1.24 ^e^	1.21 ± 0.34 ^k^	62.18 ± 12.12 ^h^
	Leaves	3.98 ± 0.34 ^b^	1.72 ± 0.56 ^b^	88.21 ± 9.76 ^c^
2	Stems	1.50 ± 0.04 ^h^	0.76 ± 0.34 ^f^	47.21 ± 11.21 ^k^
	Roots	2.87 ± 0.45 ^d^	1.18 ± 0.12 ^j^	68.21 ± 12.12 ^g^
	Leaves	4.10 ± 0.21 ^ab^	1.98 ± 0.32 ^ab^	90.17 ± 10.76 ^bc^
4	Stems	1.57 ± 0.05 ^g^	0.86 ± 0.12 ^e^	50.11 ± 5.67 ^j^
	Roots	2.92 ± 0.03 ^d^	1.27 ± 0.32 ^i^	70.82 ± 5.88 ^f^
	Leaves	4.21 ± 0.02 ^a^	2.12 ± 0.04 ^a^	98.12 ± 7.98 ^a^
6	Stems	1.92 ± 0.21 ^f^	0.97 ± 0.08 ^d^	57.12 ± 12.12 ^l^
	Roots	2.97 ± 0.11 ^de^	1.46 ± 0.12 ^h^	76.21 ± 10.12 ^e^

All results are expressed as means ± standard error of mean (SEM). N = 40. Means within columns with the same alphabets are not significantly different at *p* ≤ 0.05.

**Table 2:** Pearson’s correlation coefficients between total phenolics and total flavonoids with all parameters measured in the study.

Parameters	Pearson’s correlation coefficient (R^2^)	
	Total phenolics	Total flavonoids
1. Soluble sugar	0.971 *	0.973 *
2. H_2_O_2_	0.877 *	0.812 *
3. O_2_	0.778 *	0.912 *
4. PAL activity	0.923 *	0.901 *
5. APX	0.781 *	0.822 **
6. SOD	0.845 *	0.816 *
7. CAT	0.912 *	0.832 *
8. ORAC	0.904 *	0.956 *
9. DPPH	0.781 *	0.889 *
10. Net Photosynthesis	−0.871 *	−0.921 *
11. Stomata conductance	−0.881 **	−0.824 *
12. LOX	0.951 *	0.923 *

*, ** significant at *p* ≤ 0.05 and 0.01, respectively.

### 2.2. Soluble Sugar Profiling

The profiling of soluble sugar was influenced by ABA levels applied to *Orthosiphon stamineus* (*p* ≤ 0.01). The accumulation of carbohydrates in different parts of the plant followed a descending order with leaf > root > stem. As ABA levels increased, the concentration of soluble sugar increased (Table 1). It was found that, the concentration of sucrose and starch registered the lowest values with 0 µM ABA compared to the other treatments. In the leaves, the 0, 2, 4 and 6 µM ABA treatments resulted in the production of 79.12, 88.21, 90.17 and 98.12 mg sucrose g^−1^ dry weight, respectively. The results indicate that applications of ABA until 6 µM was able to enhance the soluble sugar content. Jaafar [34] had also observed an increase in soluble sugar content in *Capsicum annum* due to accumulation of ABA in the plant. Similar responses were also observed in maize [35] and cucumber [36] with foliar applications of ABA. Structural carbohydrates are the basic compounds required to produce carbon-based secondary metabolites in the shikimic acid pathway [37]. Ibrahim and Jaafar [38] found that increase in the production of total phenolics and flavonoids in the medicinal plant *Labisia pumila* was due to an increase in the production of carbohydrate content. Jones and Hartley [39] proposed that the increase in phenolics and flavonoids production was related to the balance between carbohydrate source and sink, the greater the source-to-sink ratio the greater would be the production of plant secondary metabolites. 

### 2.3. Antioxidant Content of H_2_O_2_ and O_2_^−^

Assay of H_2_O_2_ and O_2_^−^ was influenced by the ABA treatments (*p* ≤ 0.01; Table 3). With H_2_O_2_ the highest antioxidant activity of 2.81 µmol TE/g dry weight was recorded in the leaf receiving 6 µM ABA. This was followed by the leaf receiving 4 µM ABA (2.60 µmol TE g^−1^ dry weight), 2 µM ABA (2.43 µmol TE g^−1^ dry weight), and 0 µM ABA (2.19 µmol TE g^−1^ dry weight ), the roots receiving 6 µM ABA (1.78 µmol TE g^−1^ dry weight), 4 µM ABA (1.66 µmol TE g^−1^ dry weight), 2 µM ABA (1.52 µmol TE g^−1^ dry weight), and 0 µM ABA (1.32 µmol TE g^−1^ dry weight), and stems receiving 6 µM ABA (0.91 µmol TE g^−1^ dry weight), 4 µM ABA (0.90 µmol TE g^−1^ dry weight), 2 µM ABA (0.78 µmol TE g^−1^ dry weight) and 0 µM ABA (0.67 µmol TE g^−1^ dry weight). With O_2_^−^, the assay showed a similar pattern as H_2_O_2_. The data indicated that *O. stamineus* sprayed with ABA had high oxidative stress. There is abundant evidence to show that ROS especially H_2_O_2_ and O_2_^−^ are involved in the cellular signaling process as secondary messengers [40]. Thus, ABA mediated metabolic changes lead to an increase in endogenous ROS levels, which in turn induces antioxidative gene expression [41]. Correlation analyses shows that the increase in antioxidative properties might be up-regulated by the increase in total phenolics and flavonoids in plants receiving foliar applications of ABA (Table 3). All antioxidant properties were observed to have strong significant positive correlations with total phenolics (r^2^, H_2_O_2_ = 0.9111; O_2_^−^ = 0.887; *p* ≤ 0.05) and flavonoids (r^2^, H_2_O_2_ = 0.9111; O_2_^−^ = 0.887; *p* ≤ 0.05; Table 2). This implies that the increase in total phenolics and flavonoids with foliar ABA applications are associated with increased antioxidant capacities that allow quenching of the excited state of active oxygen species [42,43,44].

**Table 3:** Impact of abscisic acid levels on H_2_O_2,_ O_2_^−^ and PAL activity in different parts of *Orthosiphon stamineus.*

ABA (µM)	Parts	H_2_O_2_ (µmol g^−1^ fresh weight)	O_2_^−^ (µmol g^−1^ dry weight min^−1^)	PAL Activity (nm transcinnamic mg^−1^ protein^−1^ h^−1^)
	Leaves	2.19 ± 0.34 ^d^	1.09 ± 0.02 ^d^	9.21 ± 0.62 ^d^
0	Stems	0.67 ± 0.21 ^k^	0.32 ± 0.23 ^j^	2.08 ± 0.23 ^l^
	Roots	1.32 ± 0.23 ^h^	0.66 ± 0.45 ^g^	5.02 ± 0.15 ^h^
	Leaves	2.43 ± 0.31 ^c^	1.17 ± 0.32 ^c^	10.16 ± 0.82 ^c^
2	Stems	0.78 ± 0.03 ^k^	0.44 ± 0.12 ^i^	2.17 ± 0.12 ^k^
	Roots	1.52 ± 0.34 ^g^	0.72 ± 0.02 ^f^	6.23 ± 0.32 ^g^
	Leaves	2.60 ± 0.06 ^b^	1.23 ± 0.12 ^b^	13.11 ± 2.13 ^b^
4	Stems	0.90 ± 0.02 ^j^	0.56 ± 0.13 ^h^	3.21 ± 0.19 ^j^
	Roots	1.66 ± 0.21 ^f^	0.87 ± 0.01 ^e^	7.11 ± 0.34 ^f^
	Leaves	2.81 ± 0.06 ^a^	1.57 ± 0.21 ^a^	17.21 ± 1.21 ^a^
6	Stems	0.91 ± 0.12 ^i^	0.57 ± 0.11 ^h^	4.11 ± 0.6l ^i^
	Roots	1.78 ± 0.02 ^e^	0.91 ± 0.04 ^e^	8.81 ± 0.54 ^e^

Results are presented as means ± standard error of mean (SEM). N = 40. Means within columns with the same alphabets are not significantly different at *p* ≤ 0.05.

### 2.4. Phenyl Alanine Ammonia Lyase (PAL) Activity

In general, the PAL activity in *O. stamineus* was found to be highest in leaves followed by roots and stems. Also, as the concentration of ABA increased from 2 to 6 µM the PAL activity was found to increase (Table 4). In leaves the application of 6 µM ABA resulted in the highest PAL activity (17.21 nM transcinnamic mg^−1^protein h^−1^), whilst the lowest was observed in stems receiving 0 µM ABA which registered 2.08 nM transcinnamic mg^−1^ protein h^−1^. An increase in the production of total phenolics and flavonoids in the present work is attributed to an increase in PAL activity under high concentrations of ABA. Correlation analysis showed that PAL activity had significant positive relationships with total phenolics (r^2^ = 0.923; *p* ≤ 0.05) and flavonoids (r^2^ = 0.901; *p* ≤ 0.05) which indicate an up-regulation of secondary metabolite production with increased PAL activity (Table 2). The present results also indicate that under high application rates of foliar ABA, the activity of phenylalanine is increased, which simultaneously enhanced the production of secondary metabolites [45]. The increase in PAL activity with increase in ABA levels was also observed by Duan *et al.* [45] in *Cynomorium songaricum* and Poceicha *et al. *[46] in *Microdochium nivale*. These results suggest that up-regulation of production of secondary metabolites in *O. stamineus* under high levels of ABA is attributed to the increase in PAL activity. 

### 2.5. Antioxidant Enzyme Activities

Activities of the antioxidant enzymes ascorbate peroxidase (APX), catalase (CAT) and superoxide dismutase (SOD) were significantly (*p* ≤ 0.05) affected by ABA application (Table 4). The APX, CAT and SOD activities were found to be highest at the maximum ABA application of 6 µM ABA. 

**Table 4:** Impact of abscisic acid levels on antioxidant enzyme activity in different parts of *Orthosiphon stamineus*.

ABA (µM)	Parts	Ascorbate peroxidase activity (APX; mg protein^−1^ min^−1^)	Superoxide dismutase activity (SOD mg protein^−1^ min^−1^)	Catalase activity (CAT; µmol mg protein^−1^ min^−1^)
	Leaves	15.23 ± 2.34 ^d^	4.62 ± 0.11 ^d^	19.21 ± 1.27 ^d^
0	Stems	6.12 ± 0.81 ^k^	1.34 ± 0.01 ^l^	6.66 ± 2.11 ^l^
	Roots	10.11 ± 0.03 ^h^	2.98 ± 0.41 ^h^	12.17 ± 0.97 ^h^
	Leaves	17.11 ± 0.51 ^c^	4.82 ± 0.21 ^c^	20.12 ± 0.82 ^c^
2	Stems	6.11 ± 0.53 ^k^	1.52 ± 0.36 ^k^	8.27 ± 0.78 ^k^
	Roots	11.27 ± 0.14 ^g^	3.62 ± 0.15 ^g^	13.24 ± 0.11 ^g^
	Leaves	19.71 ± 0.16 ^b^	5.01 ± 0.17 ^b^	23.17 ± 0.78 ^b^
4	Stems	7.23 ± 0.42 ^j^	1.71 ± 2.11 ^j^	9.23 ± 1.19 ^j^
	Roots	13.22 ± 0.31 ^f^	3.89 ± 1.02 ^f^	16.59 ± 0.89 ^f^
	Leaves	21.62 ± 0.26 ^a^	5.27 ± 0.81 ^a^	25.12 ± 1.21 ^a^
6	Stems	9.12 ± 0.98 ^i^	1.76 ± 0.92 ^i^	10.24 ± 2.17 ^i^
	Roots	14.21 ± 1.32 ^e^	4.02 ± 1.24 ^e^	17.21 ± 0.98 ^e^

All results are presented as means ± standard error of mean (SEM). N = 40. Means within columns with the same alphabets are not significantly different at *p* ≤ 0.05.

These activities are an indication that ABA application at high doses can enhance the oxidative stress in *O. stamineus* seedlings. The increase in APX, CAT and SOD activities had a positive significant correlation with production of total phenolics and flavonoids, which indicates that an increase in oxidative stress can enhance production of secondary metabolites in *O. stamineus* seedlings under high foliar ABA applications. Induction of antioxidant enzymes was reported to be a general strategy adopted by plants to overcome oxidative stresses. The APX, CAT and SOD function as effective quenchers for ROS [47]. CAT plays an essential role in scavenging from H_2_O_2_ toxicity. The combined action of CAT and SOD converts the O^2−^ and H_2_O_2_ to water and molecular oxygen (O_2_), thus preventing cellular damage under unfavorable conditions [48]. The present study suggests that ABA application caused oxidative stress in *O. stimaneus*, and at the same time enhanced the production of secondary metabolites [49]. The study by Homa and Elham [50] had shown that production of APX, CAT and SOD was enhanced in bean seedlings after ABA application, and was followed by significant increases in H_2_O_2_. Similar observations were made in the present study where higher levels of ABA enhanced the production of antioxidant enzymes and were simultaneously followed by an increase in generation of O_2_ and H_2_O_2_. 

### 2.6. 1,1-Diphenyl-2-picryl-hydrazyl (DPPH) and Oxygen Radical Absorbance Capacity (ORAC) Assay

The purple colored DPPH is a stable free radical, which can be reduced to α,α-diphenyl-β-picryhydrazine (yellow colored) when reacted with antioxidant. The latter interrupts the free radical chain oxidation by donating hydrogen from the hydroxyl group to form a stable end product that does not initiate or propagate further oxidation of lipids [51]. Generally, DPPH antioxidant activity in *O. staminaeus* was found to be the highest on the underside of leaves, followed by the roots and the stems at all levels of ABA application (Table 5). 

**Table 5:** Impact of abscisic acid levels on antioxidant capacities in different parts of *Orthosiphon stamineus.*

ABA (µM)	Parts	ORAC (µmol Trolox equivalent g^−1^)	DPPH (µmol Trolox equivalent g^−1^)
	Leaves	65.21 ± 2.41 ^d^	20.19 ± 9.02 ^d^
0	Stems	35.67 ± 0.21 ^i^	7.72 ± 2.32 ^j^
	Roots	51.07 ± 2.23 ^g^	13.66 ± 6.45 ^h^
	Leaves	69.31 ± 10.31 ^c^	25.17 ± 8.32 ^c^
2	Stems	37.78 ± 9.03 ^k^	9.44 ± 7.12 ^k^
	Roots	55.02 ± 0.34 ^l^	15.72 ± 3.02 ^g^
	Leaves	70.60 ± 8.96 ^b^	27.84 ± 9.12 ^b^
4	Stems	40.90 ± 11.02 ^h^	10.23 ± 8.13 ^i^
	Roots	57.21 ± 10.21 ^f^	16.21 ± 2.21 ^f^
	Leaves	77.81 ± 0.06 ^a^	31.57 ± 9.21 ^a^
6	Stems	45.21 ± 0.12l ^g^	11.01 ± 2.11 ^h^
	Roots	60.23 ± 8.02 ^e^	17.81 ± 2.04 ^e^

All results are presented as means ± standard error of mean (SEM). N = 40. Means within columns with the same alphabets are not significantly different at *p* ≤ 0.05.

The DPPH antioxidant activity at 6 µM ABA was the highest (17.81–31.57 µmol TE g^−1^), followed by the 4 µM ABA (15.72–25.17 µmol TE g^−1^), 2 µM ABA (16.21–27.84 µmol TE g^−1^) and the lowest in 0 µM ABA (13.66–20.19 µmol TE g^−1^). The ORAC assay also showed the same trend as with DPPH. The antioxidant capacity measured by ORAC was much higher than DPPH. This was because ORAC and DPPH methods used different chemistry and mechanisms to measure antioxidant capacity. The ORAC applies a competitive reaction scheme, in which the substrate and the antioxidant compete for thermally generated peroxyl radicals through the decomposition of azo compounds [43,52]. However, the DPPH assay measures the capacity of an antioxidant in the reduction of an oxidant, which changes colour when reduced. The lower values for DPPH is attributed to the fact that those antioxidants that react quickly with peroxy radicals will react even slower or may not react with DPPH due to steric inaccessibility. The present results showed that the application of ABA can enhance the antioxidant capacity of *Orthosiphon stamineus* [53]. The enhancement of antioxidant capacity with application of ABA was due to enhancement of total phenolics and flavonoids with the application of ABA. From the correlation analysis (Table 2), it was apparent that total phenolics and flavonoids had significant positive relationships with DPPH and ORAC assays, implying that high antioxidant power with the application of ABA might have contributed to the higher content of gallic acid and rutin in the plant extract. Previous studies have shown that a combination of polyphenolic compounds produced a synergistic effect on DPPH and ORAC [54,55].

### 2.7. Leaf Gas Exchange

**Figure 1:**
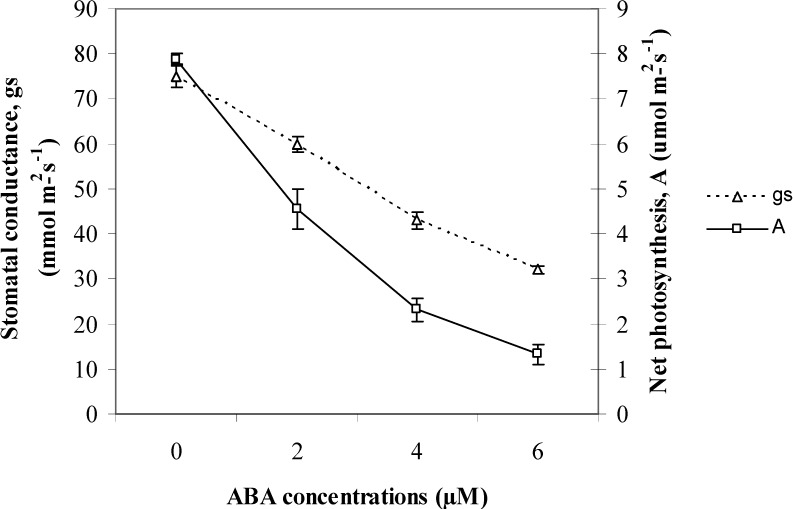
Effect of different ABA levels on leaf gas exchange in leaves of *Orthosiphon Stamineus *[N = 40; Bars represent standard error of differences between means (SEM)].

It is generally known that the accumulation of ABA can reduce stomatal conductance and net photosynthesis of plants. In the present study, it was observed that as ABA was increased from 2 to 6 µM the stomatal conductance and net photosynthesis showed a decreasing trend. The net photosynthesis with the 6 µM ABA treatment was 1.41 µmol m^−2^ s^−1^. This was 70, 211 and 451% lower compared to the treatments with 4, 2 and 0 µM ABA, respectively. The correlation analysis found that net photosynthesis had a significant negative relationship with total phenolics (r^2^ = −0.870; *p* ≤ 0.05) and flavonoids (r^2^ = −0.921; *p* ≤ 0.05) (Table 2). The present study indicates that with the application of ABA, the down-regulation of photosynthesis up-regulates the production of secondary metabolites in *O. stamineus* seedlings (Figure 1). 

This is attributed to the accumulation of ROS in *O. stamineus* seedlings. According to Bowler [56] ABA induced stomatal closure can cause a reduction in the availability of CO_2_ for photosynthesis, which may lead to the generation of ROS from the misdirection of electrons in the photosystem.

### 2.8. Lipoxygenase (LOX) Inhibitory Activity

**Figure 2:**
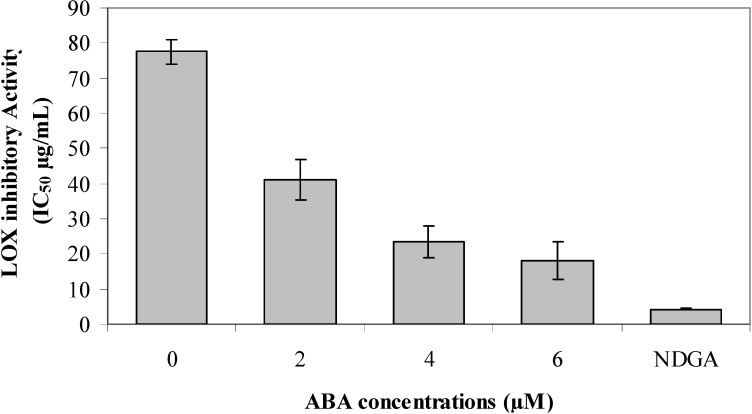
Effect of ABA levels on lipoxygenase inhibitory activity of *O. stamineus* Benth [expressed as IC_50_ (µg mL^−1^); Nordihydroguaiaretic acid (NDGA) was used as a positive standard].

LOX catalyzes dioxygenation of polyunsaturated fatty acids to yield cis, trans-conjugated diene droperoxides. LOXs are key enzymes in the biosynthesis of leukotrienes from fatty acids producing active lipid metabolites [57]. LOX is involved in provoking several inflammation-related diseases such as arthritis, asthma, cardiovascular, cancer and allergic diseases [58]. For this reason, targeting inhibitors of LOX is a promising therapeutic target for treating a wide spectrum of human diseases. Results of LOX inhibitory activity (IC_50_) are presented in Figure 2. Plants treated with 6 µM ABA extract showed the strongest ability (p < 0.05) to inhibit LOX activity (IC_50_ = 19.17 µg mL^−1^) compared to the 4 µM ABA extract (IC_50_ = 23.31 µg mL^−1^), 2 µM ABA extract (IC_50_ = 41.61 µg mL^−1^) and the 0 µM ABA extract (IC_50_ = 78.61 µg mL^−1^). However, both crude ethanol extracts possessed significantly lower (p < 0.05) LOX inhibitory activity than that of the NDGA positive standard (IC_50_ = 4.41 µg mL^−1^). The LOX inhibition in *O. stamineus* was higher than those for other common plants such as *Thespesia lampas* (600 µg mL^−1^) [59]. The current results suggest that *O. stamineus* treated with ABA had potentially high anti-LOX activity, which might be related to the high secondary metabolites content and antioxidant property of the extract [60,61].

## 3. Experimental

### 3.1. Plant Materials and Maintenance

The experiment was carried out in a glasshouse at the Faculty of Agriculture Glasshouse Complex, Universiti Putra Malaysia (longitude 101°44′N and latitude 2°58′S, 68 m above sea level) with a mean atmospheric pressure of 1.013 kPa. Stem cuttings of *O. staminaes* were propagated for two weeks in small pots and then transferred to white polyethylene bags filled with a soilless mixture of burnt rice husk and coco peat (ratio 3:1). The light intensity inside the glasshouse was from 225–1450 µmol m^−2^s^−1^. Before the experiment initiated, preliminary experiment were conducted to determine the optimum concentration of ABA to be applied to *O. staminaes*. The concentration of ABA from 0–12 μM were used. Among the treatments, the application of ABA below 7 μM have shown to significantly increased the plant dry weight and leaf number, however concentration more than 7 μM have shown to reduce the plant dry weight and leaf number. Hence the concentration < 7 μM ABA were used in this study. The exogenous ABA treatments (±*cis/trans* ABA; Sigma, St Louis, MO, USA) were applied by spraying the whole plants with 10 mL of 0, 2, 4 or 6 µM ABA per day. Abscisic acid was dissolved in water containing 315 µL L^−1^ Tween 20 as wetting agent [55]. The control solution (0 µM ABA) contained water with Tween 20. The experiment was based on a randomized complete block design with four replicates. The factor was four levels of ABA treatments (0, 2, 4, 6 μM). Each combination treatment consisted of 10 plants totaling a sum of 160 plants in the experiment. Plants were harvested for analysis at 12 weeks after planting, this is due this the plant was usually harvested three months after planting. The microclimatic conditions under the glasshouse are presented in Table 6.

**Table 6:** Microclimatic condition under the research area during 12 weeks of experiments.

Microclimate parameters	Quantification
Relative Humidity	56.14–65.32%
Light intensity	225–1450 µmol m^−2^s^−1^
Day temperature	27–31 °C
Night temperature	18–22 °C
Ambient CO_2_	372.81 µmol mol^−1^

### 3.2. Determination of Total Phenolics and Flavonoids

The method used for extraction and quantification of total phenolics and flavonoids was as described by Ibrahim and Jaafar [62]. Ground tissue samples (0.1 g) were extracted with 80% ethanol (10 mL) on an orbital shaker for 120 min at 50 °C. The mixture was subsequently filtered (Whatman™ No.1), and the filtrate was used for the quantification of total phenolics and total flavonoids. Folin–Ciocalteu reagent (diluted 10-fold) was used to determine the total phenolics content of the leaf samples. Two hundred µL of the sample extract was mixed with Follin–Ciocalteau reagent (1.5 mL) and allowed to stand at 22 °C for 5 min before adding NaNO_3_ solution (1.5 mL, 60 g L^−1^). After two hours at 22 °C, absorbance was measured at 725 nm. The results were expressed as mg g^−1^ gallic acid equivalent (mg GAE g^−1^ dry sample). For total flavonoids determination, samples (1 mL) were mixed with NaNO_3_ (0.3 mL) in a test tube covered with aluminium foil and left to stand for 5 min. Then 10% AlCl_3_ (0.3 mL) was added followed by addition of 1 M NaOH (2 mL). The absorbance was measured at 510 nm using a spectrophotometer with rutin as a standard (results expressed as mg/g rutin dry sample). For every ABA treatment, 40 plants were used as replicates.

### 3.3. Determination of Soluble Sugar

Soluble sugar was measured spectrophotometrically using the method described by Ibrahim *et al.* [63]. Samples (0.5 g) were placed in 15 mL conical tubes, and distilled water added to make up the volume to 10 mL. The mixture was then vortexed and later incubated for 10 min. Anthrone reagent was prepared by dissolving anthrone (Sigma Aldrich, St. Louis, MO, USA, 0.1 g) in 95% sulphuric acid (Fisher Scientific, Los Angeles, CA, USA 50 mL). Sucrose was used as a standard stock solution to prepare a standard curve for the quantification of sucrose in the sample. The mixed sample of ground dry sample in distilled water was centrifuged at a speed of 3,400 rpm for 10 min and then filtered to get the supernatant. A sample (4 mL) of the supernatant was mixed with anthrone reagent (8 mL) and then placed in a water-bath set at 100 °C for 5 min before the sample was measured at an absorbance of 620 nm using a model UV160U spectrophotometer (Shimadzu Scientific, Kyoto, Japan). The total soluble sugar in the sample was expressed as mg/g sucrose dry sample. About 40 plants were used as replicates for every ABA treatment.

### 3.4. Superoxide Radical (O_2_^−^) Assay

The assay for O_2_^−^ was carried out using the method of Wang *et al*. [64]. The O_2_ was generated by xanthine/xanthine-oxidase systems. Nitrite formation from hydroxylammonium chloride was determined at 530 nm in the spectrophotometer. The reaction mixture contained 1.0 mL of 65 mM Na-phosphate buffer (pH 7.8), 0.1 mL of 7.5 mM xanthine, 0.1 mL of 10 mM hydroxylammonium chloride, 0.1 mL of fruit extract, and 0.4 mL of double-distilled H_2_O. The reaction was started by addition of 0.3 mL of xanthine oxidase (containing 60 µg of protein). The total reaction volume was 2.0 mL and incubated at 25 °C for 20 min. Then, 0.5 mL was removed from the above reaction mixture, 0.5 mL of 19 mM sulfanilic acid and 0.5 mL of 1.0%-naphthylamine were added, and the mixture was shaken for 5 min. After standing at room temperature for 20 min, the optical density of the mixture was determined at 530 nm against blanks that had been prepared similarly but without plant extract. The final results were expressed as percent inhibition of O_2_ production in the presence of plant extract. The scavenging capacity of α-tocopherol at various concentrations (1 to 25 µg) on superoxide radical (O_2_) was measured and used for determining the O_2_ scavenging capacity of the plant extract. The antioxidant capacity of plant extract against the O_2_ value was expressed as µmol of α-tocopherol equivalent per gram dry weight. In each 12 experimental units, 40 plants were used as replicates.

### 3.5. H_2_O_2_ Assay

The hydrogen peroxide measurement was based on the method described by Petterson *et al*. [65]. Three hundred milligrams of the youngest, fully expanded leaf was homogenized in a cold mortar with 5 mL 5% trichloroacetic acid (TCA) containing 0.1 g activated charcoal and 0.1% polyvinyl- polypyrrolidone (PVPP). The homogenate was filtered and centrifuged at 18,000 g for 10 min. The supernatant was filtered through a Millipore filter (0.45 mm) and used in the assay. A 200-mL aliquot was brought to 2 mL with 100 mM potassium phosphate buffer (pH 8.4) and 1 mL of a colorimetric reagent was added. This reagent was prepared daily by mixing 1:1 (v/v) 0.6 potassium titanium oxalate and 0.6 mM 4-2 (2-pyridylazo) resorcinol (disodium salt). After incubating the sample solution at 60 °C for 45 min, the absorbance was measured at 508 nm. Blanks were made by replacing plant extract with 5% TCA. The antioxidant capacity of plant extract against H_2_O_2_ was expressed as µmole of ascorbate equivalent per gram dry weight. About 40 plants were used as replicates for every ABA treatment.

### 3.6. Oxygen Radical Absorbance Capacity (ORAC) Assay

The plant extracts were incubated with fluorescein as a free radical probe and AAPH as a free radical generator [66]. The kinetics of fluorescein degradation was read on a Spectra XMS Gemini microplate reader (Molecular Devices, Sunnyvale, CA, USA). A sample of 6-hydroxy-2,5,7,8-tetramethylchroman-2-carboxylic acid (Trolox) was used to generate a standard curve. The results of the antioxidant capacity of plant extracts were expressed as µmol Trolox equivalent per gram of fresh sample (µmol TE/g). For every ABA treatment, 40 plants were used as replicates.

### 3.7. DPPH Assay

The DPPH scavenging activities of freeze dried blueberries were measured using a standard method [53]. Twenty milligrams of DPPH was dissolved in 100 mL of methanol to make a DPPH stock solution. The DPPH working solutions were freshly prepared by mixing 3.5 mL DPPH stock solution and 6.5 mL methanol. The absorbance at 515 nm was measured on a Spectra max 190 microplate reader (Molecular Devices). The plant extracts (50 µL) were added to 950 µl DPPH working solution and samples were kept at room temperature in the dark for 60 min. Trolox solutions were added to DPPH working solutions as standards. The results of the DPPH scavenging activity were expressed as µmol Trolox equivalent per gram of fresh plant sample (µmol TE/g). In each 12 experimental units, 40 plants were used as replicates.

### 3.8. Phenylalanine Ammonia-Lyase (PAL) Activity

Phenylalanine-ammonia-lyase (PAL) activity was measured using the method described by Martinez and Lafuante [67]. The enzyme activity was determined by spectrophotometrically measuring the production of trans-cinnamic acid from L-phenylalanine. Enzyme extract (10 µL) was incubated at 40 °C with 12.1 mM L-phenylalanine (90 µL, Sigma) that was prepared in 50 mM Tris-HCl, (pH 8.5). After 15 min of reaction, *trans*-cinnamic acid yield was estimated by measuring increase in the absorbance at 290 nm. The standard curve was prepared by using a *trans*-cinnamic acid standard (Sigma) and the PAL activity was expressed as nM *trans*-cinnamic acid µg protein^−1^ h^−1^. In each 12 experimental units, 40 plants were used as replicates.

### 3.9. Leaf Gas Exchange Measurement

The measurements were obtained using a closed infra-red gas analyzer LICOR 6400 Portable Photosynthesis System (IRGA, Licor. Inc. Omaha, NE, USA). Prior to use, the instrument was warmed for 30 min and calibrated with the ZERO IRGA mode. Two steps are required in the calibration process: first, the initial zeroing process for the built-in flow meter; and second, zeroing process for the infra-red gas analyzer. The measurements used optimal conditions set at 400 µmol mol^−1^ CO_2_, 30 °C cuvette temperature, 60% relative humidity with air flow rate set at 500 cm^3^ min^−1^, and modified cuvette condition of 800 µmolm^−2^s^−1^ photosynthetically photon flux density (PPFD). The measurements of gas exchange were carried out between 09:00 to 11:00 a.m. using fully expanded young leaves numbered three and four from the plant apex to record net photosynthesis rate (A). The operation was automatic and the data were stored in the LI-6400 console and analyzed with the Photosyn Assistant software (Version 3, Lincoln Inc., Columbus, OH, USA). Several precautions were taken to avoid errors during measurements [68]. About 40 plants were used as replicates in each ABA treatment.

### 3.10. Antioxidant Enzyme Activity

#### 3.10.1. Preparation of Enzyme Extracts

To determine the enzymatic activity of the antioxidant proteins, a crude enzyme extract was prepared by homogenizing 500 mg of leaf tissue in extraction buffer containing 0.5% Triton X-100 and 1% polyvinylpyrrolidone in 100 mM potassium phosphate buffer (pH 7.0) using a chilled mortar and pestle. The homogenate was centrifuged at 15,000 rpm for 20 min at 4 °C. The supernatant was used in the enzymatic assays described below. For every ABA treatment, 40 plants were used as replicates.

#### 3.10.2. Ascorbate Peroxidase (APX) Activity Assay

Ascorbate peroxidase activity (APX, EC 1.11.1.11) was determined spectophotometrically by a decrease in the absorbance at 265 nm using the method of Nakano and Asada [41]. The reaction mixture contained 50 mM potassium phosphate buffer pH 7.0, 5 mM ascorbate, 0.5 mM H_2_O_2_ and enzyme extract. For every ABA treatments, 40 plants was used as replicates

#### 3.10.3. Catalase (CAT) Activity Assay

Catalase activity (CAT; EC 1.11.1.6) was determined by consumption of H_2_O_2_ using the method of Aebi [69]. The reaction mixture (3 mL) contained 50 mM potassium phosphate buffer pH 7.0, 15 mM H_2_O_2_ and 50 µL enzyme extract. The reaction was initiated by adding the H_2_O_2_. The consumption of H_2_O_2_ was monitored spectrophotometrically at 240 nm for 3 min. Enzyme activity was expressed in µM H_2_O_2_ min^−1^.

#### 3.10.4. Superoxide Dismutase (SOD) Activity Assay

The activity of SOD (EC 1.15.1.1) was determined by measuring its ability to inhibit the photoreduction of nitro blue tetrazolium (NBT) according to the method of Giannopolitis and Ries [70]. The reaction solution (3 mL) contained 50 µmol NBT, 1.3 riboflavin, 13 mmol methionine, 75 nmol EDTA, 50 mmol phosphate buffer (pH 7.8) and 50 µL enzyme extract. The reaction solution was irradiated under fluorescent light at 75 µmol m^−2^s^−1^ for 15 min. The absorbance at 560 was read against a blank (non-irradiated reaction solution). One unit of SOD activity was defined as the amount of enzyme that inhibited 50% of NBT photoreduction.

### 3.11. LOX Inhibitory Assay

Lipoxygenase (LOX) was assayed according to the method reported by Wu [71]. A mixture of a solution of sodium borate buffer (1 mL, 0.1 M, pH 8.8) and soybean LOX (10 µL, 9000 U mL^−1^) was incubated with the plant extract sample (10 µL) in a 1 mL cuvette at room temperature for 5 min. The reaction was initiated by the addition of linoleic acid substrate (10 µL, 10 mmol). The absorbance of the resulting mixture was measured at 234 nm over time at a rate of one measurement/min (6 readings). Inhibition of LOX was assessed using the following equation:

% Inhibition = 100 × (absorbance of the control − absorbance of the sample)/(absorbance of the control)

The effective concentration (µg mL^−1^) at which LOX activity is inhibited by 50% (IC_50_) was represented on a graph. Nordihydroguaiaretic acid (NDGA) was used as the positive standard. For every ABA treatment, 40 plants were used as replicates.

### 3.12. Statistical Analysis

Data were analyzed using the analysis of variance procedure in SAS version 17. Means separation was performed using Duncan multiple range test and the standard error of differences between means was calculated with the assumption that the data were normally distributed and equally replicated [72,73].

## 4. Conclusions

In conclusion, this work reveals that the use of ABA can enhance the production of primary and secondary metabolites in *O. stamineus*. The study showed increased oxidative stress (APX, CAT, SOD) at high application rates of ABA and improved production of phytochemicals (total phenolics and flavonoids). This showed that application of foliar ABA can be useful tool to enhance secondary metabolites properties of this plant.
